# Parasitic infections represent a significant health threat among recent immigrants in Chicago

**DOI:** 10.1007/s00436-020-06608-4

**Published:** 2020-02-01

**Authors:** Jesica A. Herrick, Monica Nordstrom, Patrick Maloney, Miguel Rodriguez, Kevin Naceanceno, Gloria Gallo, Rojelio Mejia, Ron Hershow

**Affiliations:** 1 Department of Medicine, Division of Infectious Diseases, Immunology, and International Medicine, University of Illinois at Chicago, 808 South Wood, M/C 735, Chicago, IL 60612 USA; 2 University of Illinois at Chicago School of Public Health, Chicago, IL USA; 3 Department of Medicine, Section of Infectious Diseases, National School of Tropical Medicine, Baylor College of Medicine, Houston, TX USA; 4 Science and Technology Institute, Federal University of São Paulo, São José dos Campos, São Paulo, Brazil

**Keywords:** Immigrant health, Strongyloidiasis, *Blastocystis*, *Toxocara*, *Giardia*

## Abstract

Parasitic infections are likely under-recognized among immigrant populations in the USA. We conducted a cross-sectional study to evaluate if such infections have health impacts among recent immigrants in Chicago and to identify predictive factors for parasitic infections. A total of 133 recent immigrants were enrolled, filling out a standardized medical questionnaire and providing blood and stool samples. Appriximately 12% of subjects (15/125) who provided a blood or stool sample for testing were found to have evidence of current or prior infection with a pathogenic parasite, of which *Toxocara* spp*.* (8 subjects, 6.4%) and *Strongyloides stercoralis* (5 subjects, 4%) were most commonly identified. Parasitic infection was more likely among subjects who had immigrated within the previous 2 years and those with a self-reported history of worms in the stool. The most useful surrogate markers identified for parasitic infections were an elevated immunoglobulin E level (seen in 46.7% (7/15) of subjects with parasitic infections and 20% (22/110) of uninfected individuals, *p* = 0.04) and the presence of *Blastocystis hominis* cysts on Ova & Parasite exam (detected in 38.5% (5/13) of subjects with parasitic infections who provided a stool sample and 5.1% (5/98) of uninfected subjects, *p* = 0.002). Our study found that parasitic infections may be common in recent US immigrants, which highlights an important health disparity among a vulnerable population that merits further study. Additionally, clinical risk factors, symptoms, and laboratory findings traditionally thought to be associated with parasites were commonly found but not predictive of infection in this study population.

**Electronic supplementary material:**

The online version of this article (10.1007/s00436-020-06608-4) contains supplementary material, which is available to authorized users.

## Introduction

Among select populations within the USA, parasitic diseases are thought to be an under-recognized public health problem (Garg et al. 2005; Hotez 2007; Hotez 2008; Hotez 2014; Hotez et al. 2012; Jariwala et al. 2017; Parise et al. 2014). Although exact numbers are not available, studies have suggested that within the USA there may be up to 300,000 people with Chagas disease (Bern et al. 2011; Bern and Montgomery 2009; Leiby et al. 2002; Manne-Goehler et al. 2016; Sarkar et al. 2010; Schmunis and Yadon 2010; Zaniello et al. 2012), 1.3–2.8 million with serological evidence of exposure to *Toxocara* spp. (Hotez 2008; Liu et al. 2018), 4 million with soil-transmitted helminths (Hotez 2008; Posey et al. 2007; Safdar et al. 2004), 1.2 million with giardiasis (Adams et al. 2017), 41,400–169,000 with cysticercosis (Hotez 2008), and approximately 8000 with schistosomiasis (Hotez 2008).

US immigrant populations are believed to be at increased risk for parasitic infections, as many immigrants come from areas where some parasites are endemic (Arena et al. 2011; Hotez 2008; Meymandi et al. 2017; Ostera and Blum 2016; Ostera et al. 2017). Whereas refugees to the USA undergo thorough screening coupled with treatment of infectious diseases prior to arrival, legal immigrants (who may have similar risk factors as refugees) are only required to undergo screening for infectious diseases of significant public health importance, such as tuberculosis. As would be expected, illegal immigrants are not screened for any infections. Upon arrival to the USA, immigrants may be more likely to have a decreased socioeconomic status and therefore frequently reside in the underserved areas within the USA (characterized by increased poverty rates, impaired access to healthcare, and a high percentage of households with suboptimal plumbing) that are thought to have an increased prevalence of parasitic infections (Hotez 2007; Hotez 2008; Hotez 2014; McKenna et al. 2017). This population thus possesses a dual risk for parasites, either acquired from their country of origin or following emigration to the USA (Parise et al. 2014). However, published prevalence estimates vary widely, and there is little data regarding the current impact of these infections in the USA.

Both diagnostic and methodologic problems have impeded research regarding parasitic infections among US immigrants. Diagnostic inaccuracies can occur because laboratory tests for parasites can have significant limitations including poor sensitivities and lack of availability in the community (Belhassen-Garcia et al. 2014; Hotez 2014; Jariwala et al. 2017; Ostera and Blum 2016; Parise et al. 2014; Posey et al. 2007; Shulman et al. 1997). Further, the primary method of diagnosis for many parasitic infections, serologic testing, has several important shortcomings, including an inability to distinguish between active and past infections, cross-reactivity between different parasites, and in many cases a reduced sensitivity due to the focal nature of some parasitic diseases (Khurana and Sethi 2017; Maddison 1991; Ndao 2009). Methodologic limitations to research studies may also partly account for these widely varying prevalence estimates (Garg et al. 2005; Hotez 2008; Hotez 2014). For example, past estimates of Chagas disease prevalence were based on testing of blood donors, which has been shown to underestimate the number of infected individuals (Bern et al. 2008). A second commonly used method creates an estimate based on the number of immigrants living in the USA and disease prevalence in endemic regions. However, infection risks among those who emigrated may be different from that of the general population within a given country (Arena et al. 2011; Bern and Montgomery 2009; Conners et al. 2016; Crum et al. 2003; Perez-Molina et al. 2012; Schmunis and Yadon 2010).

Many of the published studies evaluating health impacts of parasitic infections on immigrants either screened for only a small number of parasites or screened immigrants from a single country (Bern et al. 2011; Bern and Montgomery 2009; Ostera and Blum 2016; Ostera et al. 2017; Rapoport et al. 2015; Sarkar et al. 2010; Won et al. 2008; Zaniello et al. 2012). Improved data regarding these health impacts is badly needed as parasitic infections can have significant morbidity (Fitzpatrick et al. 2010; Hotez 2007; Hotez 2008; Hotez et al. 2012; Jariwala et al. 2017; Parise et al. 2014; Posey et al. 2007; Starr and Montgomery 2011). For example, Chagas disease causes cardiomyopathy and/or esophageal or colonic dilation in up to 20–30% of those infected (Bern et al. 2011; Moncayo and Ortiz Yanine 2006); those with toxocariasis may have an increased risk for asthma (Buijs et al. 1997; Cobzaru et al. 2012; Kanobana et al. 2013; Walsh 2011); and intestinal parasite infection has been associated with delayed cognitive development and impaired nutrition in some studies (Ezeamama et al. 2005; Yap et al. 2012). Parasitic infections have also been shown to increase the severity of illnesses due to other infectious diseases such as tuberculosis and HIV (Garg et al. 2005).

This study was conducted with the overall aim of assessing if parasitic infections are a significant health problem among recent immigrants, as currently there are few studies that have addressed this question. As many of the symptoms of parasitic infections are nonspecific, an additional objective of our study was to identify if there are symptoms, signs, or laboratory tests that could be used in this population as predictors of the presence of parasites.

## Materials and methods

### Compliance with ethical standards

The study was approved by the Institutional Review Board of the University of Illinois at Chicago, and written informed consent was obtained from all participants. For Spanish-speaking individuals, consent was obtained using consent and/or assent forms translated into Spanish. Subjects who spoke languages other than English or Spanish had the consent form explained to them in their language of preference (using an interpreter). For enrolled individuals between the ages of 10 and 18, the subject signed an assent form, and a parent or guardian signed a parental permission form. Each participant received $40 to compensate them for their time. Treatment for parasitic infections was not a part of the study, but all subjects were offered the possibility of seeing one of the study authors (JH) in clinic free of charge to discuss treatment options.

### Study design

Patients were enrolled for this cross-sectional study between November 2014 and July 2016. Subjects were recruited through collaboration with local community organizations that provide services to immigrants and through a mass email sent to students enrolled at the University of Illinois at Chicago. Subjects were also recruited from community health clinics as long as they were accessing the healthcare system for reasons clearly unrelated to a parasitic infection (e.g., patients presenting for routine physical exams). Subjects were eligible for inclusion if they were ages 10–80 and an immigrant living in the US mainland for less than 5 years and were originally from Africa, Asia, South America, Central America, Mexico, the Caribbean islands, or Puerto Rico. Although they are US citizens and not immigrants from non-US territories, Puerto Ricans were specifically included in this study because previous research suggested that parasitic infections were common among the Puerto Rican population in Chicago (Winsberg et al. 1975). The 5-year time point for living in the USA was chosen based on the average expected life-span of hookworms, *Ascaris lumbricoides*, and *Trichuris trichiura*, a primary focus of our investigations (Jourdan et al. 2017). Subjects were excluded from the study if they (1) were currently pregnant, as the immunosuppression of pregnancy may alter the presentation and one of the aims of the study was to identify factors predictive of parasitosis, or (2) had taken antiparasitic medications since moving to the continental USA.

Upon enrollment, a standardized questionnaire (Online Resource – Supplemental Appendix) was administered to collect information regarding demographics, risk factors for parasitic infections, and symptoms. Participants then provided clinical samples to be tested for evidence of parasitic infections as described below.

### Laboratory testing

Whole blood for a complete blood count was collected in an ethylenediaminetetraacetic acid (EDTA) tube and processed in the clinical laboratory at the University of Illinois at Chicago (BXH 800 or BXH 1600, Beckman Coulter, Brea, CA) within 12 h. Serum samples were collected in a serum separator tube (SST) and centrifuged within 1 h of collection. A portion of the supernatant was sent for measurement of immunoglobulin E (IgE) level (Quantitative ImmunoCAP® Fluorescent Enzyme Immunoassay, Arup Laboratories, Salt Lake City, UT). The remainder of the supernatant was stored at − 80 °C until samples were shipped as a batch to the Centers for Disease Control and Prevention, where serologic testing was performed for infections endemic to each subject’s country of origin (Online Resource Table 1).

Antibody responses to cysticercosis, strongyloidiasis, and toxocariasis were determined using a multiplex bead-based assay, MBA (Anderson et al. 2015; Hernandez-Gonzalez et al. 2017; Rascoe et al. 2015). For cysticercosis, three recombinant antigens (rGP50, rT24H, and sTs18var1) were used; one recombinant antigen was used for both strongyloidiasis (Ss-NIE-1) and toxocariasis (rTc-CTL-1). The assays were conducted and interpreted according to previously published methods (Anderson et al. 2015; Hernandez-Gonzalez et al. 2017; Rascoe et al. 2015).

Antibody against *T. cruzi* antigens was detected using a commercial kit (Weiner Lab, Argentina) and by following the company protocol instructions (Affranchino et al. 1989; Chagatest: ELISA recombinante v. 3.0. Rosario n.d.; da Silveira et al. 2001). All serum specimens were initially tested by FAST-ELISA using *Schistosoma mansoni* adult microsomal antigen and then by a species-specific immunoblot appropriate to the subject’s country of origin according to previously published methods (Hancock and Tsang 1986; Tsang and Wilkins 1991; Tsang and Wilkins 1997).

Patients also provided a stool sample for standard Ova & Parasite (O&P) direct microscopy (Arup Labs Qualitative Concentration/Trichrome Stain/Microscopy, Salt Lake City, UT) and a multi-parallel quantitative polymerase chain reaction (qPCR) which tests for eight common gastrointestinal pathogens (*Ancylostoma*, *Ascaris*, *Cryptosporidium*, *Entamoeba*, *Giardia*, *Necator*, *Strongyloides*, and *Trichuris*). Only one stool sample was requested for O&P due to our desire to compare O&P directly with qPCR and because the yield of collecting multiple samples for O&P exam for any new identifiable pathogens has been shown to be low in immigrant populations (Bass et al. 1992). The stool samples for qPCR were frozen without fixatives at −80 °C and shipped as a batch to the Baylor College of Medicine where the DNA was extracted using the MP FastDNA for Soil Kit (MP Biomedicals, Solon, OH) and qPCR were conducted according to previously published methods (Cimino et al. 2015; Mejia et al. 2013; Weatherhead et al. 2017).

### Statistical analysis

Statistical analyses were performed using Prism 7 (GraphPad, San Francisco, CA) and SAS (Cary, NC). Data were summarized by using frequency with percentage for categorical variables. For continuous variables, geometric mean with standard deviation or median with range or interquartile range was used. For categorical variables, prevalence ratios (PR) were used to evaluate relationships between covariates and the main outcome (the presence of a parasitic infection), and Fisher’s exact test or chi-squared test were used to determine significance. Mann-Whitney U test was used to evaluate for associations between continuous variables and the presence of a parasite. Correlations were calculated using Spearman’s rank correlation coefficient. Kappa values were used to compare results from stool O&P exam to qPCR testing.

## Results

### Patient characteristics

Seven hundred and thirty-eight people were approached about the study, and 133 were enrolled (Online Resource Fig. 1). All enrolled subjects filled out the study questionnaire (unedited results shown in Online Resource Table 2); 94% of enrolled subjects (125/133) provided blood, and 85% (113/133) provided stool samples for testing. The mean age of enrolled subjects was 32 years, and 45.1% (60/133) were male (Table 1). Participants came from 28 different countries. As shown in Table 1, many subjects reported having been exposed to risk factors for parasitic infection.

**Table 1:** Demographics, symptoms, and laboratory results of 133 enrolled subjects

Characteristic	Results^a^
Age, mean years (standard deviation)	32 (17.3)
Gender, male/female, N (% male)	60:73 (45.1)
Country/region of origin, N (%)	
Asia (excluding India) Mexico India Central/South America Africa Middle East USA (Puerto Rico)^b^	33 (24.8) 30 (22.6) 26 (19.5) 22 (16.5) 13 (9.8) 5 (3.8) 4 (3)
Mean number of years in school (standard deviation)	9.9 (5.2)
Annual household income in US dollars, N (%)	
< 20,000 20,000–40,000 40,000–60,000 60,000–100,000 > 100,000	95 (71.4) 30 (22.6) 5 (3.8) 2 (1.5) 1 (0.8)
Length of time living in the continental USA^c^, mean years (range, standard deviation in years)	2 (10 days–5 years, 1.6)
Raised in rural environment, N (%)	25 (18.8)
Exposure history prior to immigration, number yes (%)	
Frequently walked barefoot Close contact with animals Used a well as source of drinking water Bathed in streams or ponds Lived in a house with a thatched roof	76 (57.1) 65 (48.9) 65 (48.9) 58 (43.6) 25 (18.8)
Treatment with antiparasitic medication prior to emigration, number yes (%)	46 (34.6)
Symptoms reported by the patient on the day of study enrollment^d^, N (%)	
Gastrointestinal Constitutional Musculoskeletal Dermatologic Pulmonary Cardiovascular Allergic	32 (24.1) 25 (18.8) 25 (18.8) 13 (9.8) 12 (9) 10 (7.5) 3 (2.3)
Immunoglobulin E (IgE) results	
Subjects with elevated Immunoglobulin E, N (%) Immunoglobulin E level, IU/mL, median (range, standard deviation)	29/125^e^ (23.2) 57 (3–7732, 706)
Absolute eosinophil count (AEC) results	
Subjects with elevated absolute eosinophil count, N (%) Absolute eosinophil count, median cells/μl (range, standard deviation)	12/123^f^ (9.8) 200 (0–1600, 240)
Pathogenic parasitic infection, N (%)	15/125^g^ (12)

^a^Unedited results from all enrolled subjects are shown in Online Resource Table 2; *N* number of subjects

^b^Although they are US citizens and not immigrants from non-US territories, Puerto Ricans were specifically included in this study because previous research suggested that parasitic infections were common among the Puerto Rican population in Chicago

^c^Potential range (due to enrollment criteria) 0–5 years

^d^Gastrointestinal symptoms included heartburn, difficulty or pain with swallowing, abdominal pain, diarrhea, and nausea/vomiting; constitutional symptoms included weight loss, fatigue, fevers, and weakness; musculoskeletal symptoms included pain and stiffness in the joints or myalgias; dermatologic included itching, hives, or rash; pulmonary symptoms included wheezing, shortness of breath, and cough; cardiovascular included chest pain, palpitations, or irregular heart beat; and allergic symptoms included those who responded “yes” to the question “do you currently have any symptoms of seasonal allergies”

^e^Of the 125 subjects who underwent a blood draw, normal value of IgE defined as < 696 IU/mL for subjects 10–12 years old, < 629 for those ages 13–15, < 537 for those ages 16–17, and < 214 IU/mL in adults

^f^For two of the 125 subjects who underwent a blood draw, a complete blood count was unable to be performed due to clotting of the blood sample, normal AEC defined as < 500 cells/UL

^g^Of 125 subjects who provided a clinical sample (blood or stool) for testing

### Laboratory testing

The median IgE among the 125 subjects from whom blood samples were obtained was 57 IU/mL (range 3–7732), and 23.2% of subjects (29/125) had elevated IgE levels. Twelve subjects (9.8%) were found to have eosinophilia, and the median absolute eosinophil count (AEC) level was 200 cells/UL (range 0–1600 cells/UL, Table 1).

### Parasitic infections

Twelve percent (15/125) of subjects who provided a clinical sample for testing were found to have evidence of current or prior infection with a pathogenic parasite species. As shown in Table 2, the most common infections identified were *Toxocara* spp*.*, seen in eight subjects (6.4%), followed by *S. stercoralis* (five subjects, 4%, Table 2).

**Table 2:** Parasitic infections diagnosed among recent immigrants living in Chicago

Parasitic infections	Number of subjects infected (%)	
Soil-transmitted helminths		
*Toxocara* spp*.*	8 (6.4)^a^	
*Strongyloides stercoralis*	5 (4)^b^	
*Trichuris trichiura*	2 (1.8)^c^	
*Ascaris lumbricoides*	1 (0.9)^c^	
Protozoa		
*Giardia duodenalis*	4 (3.5)^d^	
Nonpathogenic or disputed pathogenicity species^e^		
*Blastocystis hominis*	10 (9)	
*Endolimax nana*	4 (3.6)	
*Dientamoeba fragilis*	3 (2.7)	
*Iodamoeba bütschlii*, *Entamoeba coli*, *Entamoeba hartmanni*	1 each (0.9)	
Number of subjects with multiple pathogens	3 (2.4)^f^	
Comparison of Ova & Parasite Exam to qPCR for pathogenic species^g^	qPCR positive	qPCR negative
Ova & Parasite positive	1	0
Ova & Parasite negative	7	107

^a^Of 125 subjects who provided blood samples for testing, all 8 subjects were diagnosed based on a positive serologic test

^b^Of 125 subjects who provided blood and/or stool samples for testing, 1 subject tested negative on O&P but positive on both serology and stool qPCR, 3 subjects had a positive serologic test but negative O&P and qPCR, and 1 subject had a positive serology but did not provide a stool sample for testing

^c^Diagnosed based on a positive stool qPCR test, of 113 subjects who provided a stool sample for qPCR testing

^d^Of 113 subjects who provided a stool sample for testing, 1 subject tested positive on both qPCR and O&P tests, and 3 subjects tested positive on qPCR but negative on O&P

^e^Of 111 subjects who returned a stool sample for O&P testing, all infections diagnosed based on a positive on O&P exam as these nonpathogenic or disputed pathogenicity organisms were not tested for on qPCR

^f^Of 125 subjects who provided blood and/or stool samples for testing, one subject had positive stool qPCR tests for both *Ascaris* and *Trichuris* and a positive serologic test for *Toxocara*, 1 subject had positive stool qPCR tests for *Trichuris* and *Giardia* as well as positive serologic testing for *Toxocara*, and the third subject had positive serologic testing and stool qPCR for *S. stercoralis* and positive serologic testing for *Toxocara*

^g^*p* value = 0.07, sensitivity of O&P compared to qPCR 12.5%, specificity 100%, positive predictive value (PPV) 100%, negative predictive value (NPV) 93.9%, kappa 0.21

### Outcome assessment

There were no significant differences in symptoms, exposures, or laboratory results between those found to be mono-infected with each of the different parasite species detected. Consequently, for our primary analysis, we compared uninfected individuals to subjects with evidence for any pathogenic parasitic infection with infected subjects treated as a single group.

A large number of subjects (10/111 subjects who provided a sample for O&P testing, 9%) were found to have *B. hominis*. Although the pathogenicity of *B. hominis* remains controversial (Andersen and Stensvold 2016; Roberts et al. 2014), infection with this organism in our sample was not associated with any significant differences in symptoms or laboratory values compared to the uninfected group. Accordingly, in our analysis *B. hominis* was not included as a pathogenic parasitic infection.

### Demographic characteristics and exposure histories of subjects with parasitic infections

Nearly all of the subjects found to have evidence of a pathogenic parasite (14/15, 93.3%) had lived in the continental USA for 2 years or less (compared to 70/110 of those who were uninfected, 63.6%, PR 6.8, *p* = 0.02, Table 3). This history was therefore a very sensitive, though not specific, marker for parasitic infections (sensitivity 93.3%, specificity 36.4%, PPV 16.7%, NPV 97.6%). No other demographic variables, including age, gender, recruitment site, or region of origin differed significantly between those with and without infections (Online Resource Table 3).

**Table 3:** Findings associated with the presence of a parasitic infection

Demographics	Uninfected *N* = 110	Parasitic infection^a^ *N* = 15	Prevalence ratio^b^ (95% confidence interval)	*P* value
Length of time living in the USA, N (%)				
> 2 years < 2 years	40 (36.4) 70 (63.6)	1 (6.7) 14 (93.3)	Reference 6.8 (0.9–50.2)	0.02
Mean age (standard deviation)	31 (16.4)	37.5 (21.6)	1.0 (1.0–1.1)	0.2
Gender, N (%)				
Female Male	58 (52.7) 52 (47.3)	8 (53.3) 7 (46.7)	Reference 1.0 (0.4–2.5)	0.99
Childhood exposures (in country of origin), number of respondents reporting exposure (%)				
Self-reported history of seeing worms in the stool	18 (16.4)	8 (53.3)	4.4 (1.7–10.9)	0.003
Raised in rural environment	19 (17.3)	3 (20)	1.2 (0.4–3.8)	0.73
Laboratory results				
IgE, number elevated^c^ (%)	22 (20)	7 (46.7)	3.5 (1.4–8.8)	0.04
AEC, number elevated^d^ (%)	9 (8.3)^e^	3 (20)	2.4 (0.8–7.2)	0.2
*Blastocystis hominis* cysts present on O&P exam	5 (5.1^f^)	5 (38.5^g^)	6.3 (2.5–15.7)	0.002

*N* number of subjects

^a^Not including nonpathogenic or disputed pathogenicity organisms

^b^Prevalence ratio comparing the frequency of the listed demographic variable, symptom, or exposure in those with versus those without evidence of a parasitic infection

^c^Normal value of IgE < 696 IU/mL for subjects 10–12 years old, < 629 for those ages 13–15, < 537 for those ages 16–17, and < 214 IU/mL in adults

^d^Normal AEC < 500 cells/UL

^e^Of 108 subjects in the uninfected group who were tested for a CBC (as for 2 patients who underwent a blood draw, the CBC was unable to be performed due to clotting of blood in the tube)

^f^Of 98 subjects in the uninfected group who provided a stool sample for O&P exam

^g^Of 13 subjects in this group who provided a stool sample for O&P exam

A self-reported history of ever having seen worms in the stool was also associated with evidence of current or prior parasitic infection (8/15, 53.3% in the infected group reported this history versus 18/110, 16.4%, in the uninfected group, *p* = 0.003, PR 4.4, Table 3). This history appeared to be a nonspecific marker and was equally common among those diagnosed with enteric parasites compared to those with non-enteric parasites (*p* value = 0.8). Other classic risk factors for parasitic infections (close contact with animals, etc.), although commonly reported among study participants, did not differ between groups (all *p* values > 0.05, Online Resource Table 3).

In the year prior to study enrollment, subjects with evidence of parasitic infections had experienced an overall increased number of physical complaints (median 8) compared to those with no evidence of parasites (4, *p* = 0.02). However, symptoms typically thought of as indicative of parasitic infections (rash, pruritus, etc.) did not differ significantly between the groups (all *p* values > 0.05 for symptoms experienced both the day of study enrollment and within the previous year, Online Resource Table 3).

### Laboratory values in subjects with evidence of parasitic infections

Both the median IgE level (249 IU/mL) and the proportion of subjects with an increased IgE level (7 of 15 subjects, 46.7%) were significantly increased in subjects with evidence for parasitic infections compared to those who were uninfected (median IgE 52 IU/mL, 22/110, 20%, subjects with elevated IgE level, both *p* values < 0.05, Table 3 and Online Resource Table 4). Increases in IgE were not associated with a history of seasonal allergies and/or asthma (all *p* values = NS). The difference in median IgE levels between groups remained statistically significant when subjects < 18 years old were excluded from analysis (as normal values for IgE are higher in this group). Although an elevated IgE level was a relatively specific finding for parasitic infections (specificity 80%, NPV 92%), this finding was not a sensitive marker of infection (sensitivity 47%, PPV 24%).

The median AEC did not differ between those with and without evidence of a parasitic infection (Online Resource Table 4). Despite a low sensitivity for parasites (20%, PPV 25%), however, eosinophilia was relatively specific to subjects with evidence of parasitic infection (specificity 91.8%, NPV 89.4%).

The presence of cysts of *B. hominis* on O&P exam was strongly associated with an increased likelihood for infection with a pathogenic parasite species. About 39% (5/13) of subjects in the parasite positive group who returned a stool sample had a positive O&P for *Blastocystis*, compared to 5% (5/98) tested for O&P exam in the uninfected group (*p* = 0.002, PR = 6.3, Table 3).

## Discussion

Despite the fact that many US immigrants come from areas where parasitic infections are endemic, it is not known if these infections are currently an important health issue among immigrant populations. In response to this deficit, we conducted a comprehensive evaluation for the presence of a broad array of parasitic infections in recent immigrants in Chicago. We found that 12% of recent immigrants in our sample had evidence of prior or current infection with a pathogenic parasite species.

Although limited by the small number of subjects found to have evidence of parasitic infection, our study still identified a few key factors which were significantly more likely in subjects with evidence of parasites: a self-reported history of having previously observed worms in the stool, having immigrated within the past 2 years, and reporting a relatively high number of nonspecific physical ailments within the previous year. Our results also highlight that clinical risk factors (contact with animals and frequently walking barefoot), symptoms (including urticaria, rash, and diarrhea), and laboratory findings (eosinophilia) traditionally thought to be associated with parasites were commonly found but not predictive of infection in this study population. If confirmed in a larger sample, these results suggest that practitioners should have a high degree of suspicion for parasitic infections when evaluating recent immigrants regardless of the presence or absence of symptoms or laboratory abnormalities.

Our study results showed that a high percentage of recent immigrants had evidence of current or prior infection with a few key pathogenic parasites, including *S. stercoralis*, *Toxocara* spp*.*, and *G. lamblia*. Positive serologic testing for *S. stercoralis* has been shown to be associated with a high likelihood of active ongoing infection and risk for developing hyperinfection syndrome (Posey et al. 2007; Vadlamudi et al. 2006). The rate of seropositivity for strongyloidiasis in our sample was 4%. This may have clinical implications because a study evaluating the cost-effectiveness of empiric treatment versus a test-and-treat strategy found that if the prevalence of *Strongyloides* infection is greater than 2% in a community, then the presumptive treatment strategy is more cost-effective (Muennig et al. 2004). However, whether this strategy would still be cost-effective today is not clear and merits further study. Additionally, in areas with a large number of West African immigrants, this treatment strategy would not be possible due to concerns of coinfection with *Loa loa.*

Our results highlight the limitations of current diagnostic tests for parasitic infections. For example, the sensitivity of O&P exam compared to qPCR testing (which is not yet universally available) for pathogenic parasites was very poor. While this could represent false positive qPCR tests, previous use of these tests in other studies has indicated a low rate of false positives (Cimino et al. 2015; Hochberg et al. 2011; Mejia et al. 2013; Winsberg et al. 1975). An additional limitation of diagnostics for parasites is that, for many parasitic infections, serologic testing is the primary commercially available means of diagnosis. Positive serologic testing is not proof of an active infection, so the utility of this information in treating an individual patient is limited.

Similar to prior studies of immigrant populations (Belhassen-Garcia et al. 2014; Schulte et al. 2002), we found that a relatively high percent of subjects in our study had eosinophilia and/or elevated IgE levels. IgE appeared to be a slightly better marker for chronic parasitic infection in this sample than eosinophilia. Prior studies have shown that for some parasites (such as *Toxocara*), specific anti-parasite IgE may be an even better diagnostic marker (Magnaval et al. 2006). However, neither the AEC nor the IgE had an adequate sensitivity to merit recommending their use for routine screening purposes. Although eosinophilia has long been thought of as a hallmark of helminth infection, several previous studies have also failed to show any correlation between the two (Asgary et al. 2011; Dawson-Hahn et al. 2010).

A large number of subjects in our study demonstrated cysts of *B. hominis* on O&P exam. The association between *B. hominis* and clinical disease has been controversial. Various studies have shown that subjects with *B. hominis* may have an increased likelihood of irritable bowel syndrome and that symptomatic patients with this parasite may experience a beneficial effect from treatment with antiparasitic medications (Andersen and Stensvold 2016; Coyle et al. 2012). In our study, however, infection with *B. hominis* was not associated with any increase in symptoms. Interestingly, though, subjects with *B. hominis* cysts on O&P exam were more likely to also test positive for pathogenic parasitic infections. Therefore, our results agree with those of several recent studies and suggest that the primary import of *B. hominis* infection may be serving as a surrogate marker for the presence of fecal-oral transmission (Coyle et al. 2012; Roberts et al. 2014; Turkeltaub et al. 2015).

Our study had several limitations. The small number of subjects found to have parasitic infections limited our ability to identify risk factors associated with these infections. However, our sample size was necessitated by the extensive testing performed on each subject and was also similar to those of previous studies of immigrant health (Hochberg et al. 2011; Loutfy et al. 2002). Furthermore, the fact that our sample was very heterogeneous and subjects enrolled in the study came from many different countries could have reduced our ability to find associations between clinical factors and the presence of infection. We chose to include subjects from many different countries because the goal of the study was to evaluate if parasitic infections are a potential health problem within immigrant communities in general.

Our study is one of the only recent studies to evaluate if parasitic infections currently pose health threats among immigrant communities. Our results provide an argument for further investigation of the health impacts of these infections in the immigrant community. Although our sample size was limited, a strength of our study is that we conducted a comprehensive evaluation for parasitic infections including testing of both stool and serum samples with novel molecular tests. Our results suggest that as many as 12% of recent immigrants in the community may have evidence of current or previous infection with a pathogenic parasitic species. From a public health perspective, it is important to note that the infections identified in the current patient sample are not typically spread from person to person. If confirmed in a larger study, these results present an important health disparity among a vulnerable underserved population in the USA. This health disparity has persisted despite the presence of effective, safe, and well-tolerated antiparasitic medications capable of treating each of the identified pathogens in our sample (Muennig et al. 2004; Posey et al. 2007).

## Electronic supplementary material

ESM 1 (DOCX 26 kb)

ESM 2 (PDF 59 kb)

ESM 3 (PDF 19 kb)

## Data Availability

All data generated or analyzed during this study are included in this published article and its supplementary information files.
