# Supplementary material for: Parasitic infections represent a significant health threat among recent immigrants in Chicago
Source: Parasitol Res. 2020 Feb 1;119(3):1139–48. doi: 10.1007/s00436-020-06608-4 (PMC7075846; doi:10.1007/s00436-020-06608-4)
Supplement: Supplementary file 1 — (DOCX 26 kb) [file 436_2020_6608_MOESM1_ESM.docx]

**Online Resource Table 1. Serologic testing performed based on subjects’ countries of origin.**

| **Country** | **Serologic Testing Performed^a^** |
| --- | --- |
| Afghanistan | No additional testing done |
| Algeria | *Schistosoma spp. (Schisto FAST test) and Schistosoma haematobium* |
| Bangladesh | *Schistosoma spp. (Schisto FAST test)* |
| Belize | *Schistosoma* spp. (Schisto FAST test)*,* and Chagas |
| Brazil | *Schistosoma* spp*.* (Schisto FAST test), and Chagas |
| Burma | No additional testing done |
| Cameroon | No additional testing done |
| China | Schistosoma spp. (Schisto FAST test) and *Schistosoma japonicum* |
| Columbia | No additional testing done |
| Ecuador | No additional testing done |
| Ethiopia | No additional testing done |
| Guatemala | *Schistosoma* spp. (Schisto FAST test) and Chagas |
| Honduras | No additional testing done |
| India | Schistosoma spp. (Schisto FAST test) |
| Iran | Schistosoma spp. (Schisto FAST test) and *Schistosoma haematobium* |
| Mexico | *Schistosoma* spp. (Schisto FAST test) and Chagas |
| Mongolia | *Schistosoma* spp. (Schisto FAST test) |
| Nepal | No additional testing done |
| Nigeria | No additional testing done |
| Pakistan | Schistosoma spp. (Schisto FAST test) and *Schistosoma haematobium* |
| Peru | *Schistosoma* spp. (Schisto FAST test) and Chagas |
| Sri Lanka | *Schistosoma* spp. (Schisto FAST test) |
| Taiwan | Schistosoma spp. (Schisto FAST test), *Schistosoma mansoni,* and *Schistosoma japonicum* |
| Turkey | Schistosoma spp. (Schisto FAST test) and *Schistosoma haematobium* |
| UAE | Schistosoma spp. (Schisto FAST test), *Schistosoma mansoni*, and *Schistosoma haematobium* |
| USA (Puerto Rico) | Schistosoma spp. (Schisto FAST test) and Chagas |
| Venezuela | *Schistosoma* spp. (Schisto FAST test)*,* and Chagas |

1. Serologic testing performed at the Centers for Disease Control and Prevention.

Subjects from ALL countries had serologies tested for neurocysticercosis, *Toxocara* spp.*,* and *Strongyloides stercoralis*. Additional serologic testing was performed based on the subjects' countries of origin as detailed in the table above.

**Online Resource Table 3. Symptoms and exposures evaluated for an association with the presence of a parasitic infection**

| **Demographics** | **Uninfected**  **N = 110** | **Parasitic Infection^a^**  **N = 15** | **Prevalence ratio^b^**  **(95% Confidence Interval)** | **p-value** |
| --- | --- | --- | --- | --- |
| **Country/Region of origin, N (%)**  Mexico  Asia (excluding India)  India  Central/South America  Africa  Middle East  USA (Puerto Rico)^c^ | 26 (23.6)  24 (21.8)  21 (19.1)  18 (16.4)  12 (10.9)  5 (4.5)  4 (3.6) | 2 (13.3)  8 (53.3)  2 (13.3)  2 (13.3)  1 (6.7)  0 (0)  0 (0) | Reference  3.5 (0.8-15.1)  1.2 (0.2-8)  1.4 (0.2-9.1)  1.1 (0.1-10.8)  Undefined  Undefined | 0.28 |
| **Years of schooling**  > 12  9-12  < 8 | 56 (51.9)^d^  28 (25.9)  24 (22.2) | 5 (33.3)  4 (26.7)  6 (40) | Reference  1.5 (0.4-5.7)  2.4 (0.7-7.9) | 0.27 |
| **Annual household income, US dollars**  > 20,000  < 20,000 | 34 (30.9)  76 (69.1) | 2 (13.3)  13 (86.7) | Reference  2.6 (0.6-11.1) | 0.2 |
| Childhood exposures (in country of origin), number of respondents reporting exposure (%) |  |  |  |  |
| Used a well as source of drinking water | 49 (44.5) | 11 (73.3) | 3.0 (1.0-8.9) | 0.05 |
| Frequently walked barefoot | 60 (54.5) | 11 (73.3) | 2.1 (0.7-6.2) | 0.27 |
| Close contact with animals | 51 (46.4) | 7 (46.7) | 1.0 (0.4-2.6) | 0.99 |
| Bathed in streams or ponds | 47 (42.7) | 7 (46.7) | 1.2 (0.4-3.0) | 0.79 |
| Lived in a house with a thatched roof | 20 (18.2) | 3 (20) | 1.1 (0.3-3.6) | 0.99 |
| Treated with antiparasitic medication (prior to emigration) | 38 (34.5) | 7 (46.7) | 1.6 (0.6-4.0) | 0.4 |
| Symptoms reported by the patient on the day of study enrollment^e^ |  |  |  |  |
| Gastrointestinal | 26 (23.6) | 3 (20) | 0.8 (0.3-2.7) | 0.99 |
| Constitutional | 17 (15.5) | 6 (40) | 3 (1.2-7.5) | **0.03** |
| Musculoskeletal | 18 (16.4) | 6 (40) | 2.8 (1.1-7.1) | **0.04** |
| Dermatologic | 11 (10) | 1 (6.7) | 0.7 (0.1-4.7) | 0.99 |
| Pulmonary | 10 (9.1) | 1 (6.7) | 0.7 (0.1-5.1) | 0.99 |
| Cardiovascular | 7 (6.4) | 2 (13.3) | 2 (0.5-7.5) | 0.29 |
| Allergic | 2 (1.8) | 0 (0) | Undefined | 0.99 |

N – number of subjects; a – Not including non-pathogenic or disputed pathogenicity organisms; b – prevalence ratio comparing the frequency of the listed demographic variable, symptom, or exposure in those with versus those without evidence of a parasitic infection; c - although they are U.S. citizens and not immigrants from non-US territories, Puerto Ricans were specifically included in this study because previous research suggested that parasitic infections were common among the Puerto Rican population in Chicago; d – 108 of the 110 subjects without parasitic infections responded to this question; e - gastrointestinal symptoms included: heartburn, difficulty or pain with swallowing, abdominal pain, diarrhea, nausea/vomiting; constitutional symptoms included: weight loss, fatigue, fevers, and weakness; musculoskeletal symptoms included pain and stiffness in the joints or myalgias; dermatologic: itching, hives, or rash; pulmonary symptoms: wheezing, shortness of breath, cough; cardiovascular: chest pain, palpitations, or irregular heart beat; and allergic symptoms included: those who responded “yes” to the question “do you currently have any symptoms of seasonal allergies.”

**Online Resource Table 4. Laboratory values evaluated for an association with parasitic infection.**

| **Number of subjects with abnormal laboratory test** | **Uninfected,**  **N (%)** | **Parasitic infection^a^, N (%)** | **P-value** |
| --- | --- | --- | --- |
| Immunoglobulin E^b^, Median (IQR), IU/mL  Infected with 1 parasite  2+ parasitic infections | 52 (26 - 177) | 249 (67 - 480)  111 (50.5–311)  662 (267–1097) | **0.005** |
| Absolute Eosinophil Count^c^, Median (IQR), cells/UL | 200 (100 - 200) | 200 (100 - 400) | 0.1 |
| Hemoglobin, mg/dL, mean (standard error) | 14.4 (0.2) | 14.1 (0.3) | 0.6 |

N – number; a – excluding non-pathogenic or disputed pathogenicity species; IQR – interquartile range; b - normal value of IgE < 696 IU/mL for subjects 10-12 years old, < 629 for those ages 13-15, < 537 for those ages 16-17, and < 214 IU/mL in adults; c - normal AEC < 500 cells/UL
