# Supplementary material for: Parasitic infections represent a significant health threat among recent immigrants in Chicago
Source: Parasitol Res. 2020 Feb 1;119(3):1139–48. doi: 10.1007/s00436-020-06608-4 (PMC7075846; doi:10.1007/s00436-020-06608-4)
Supplement: Supplementary file 2 — (PDF 59 kb) [file 436_2020_6608_MOESM2_ESM.pdf]

Study Questionnaire.

Please answer the following questions to the best of your ability.

1. What is your age? _______ years

2. What is your gender: ____ Male ____ Female

3. Where were you born (City and Country)? Country:___________

City: ____________

For the following questions, please circle the right answer.

3.a. Did you grow up in the city or in the country? City Country

3.b. Do you recall drinking well-water as a child? Yes No

3.c. Did you live in a house with a thatched roof? Yes No

3.d. Did you spend a large amount of time around animals as a child?

Yes No

If so, which animals (please list):

3.e. Do you recall bathing in streams or ponds as a child?

Yes No

3.f. Do you recall frequently walking barefoot as a child?

Yes No

4. How long have you lived in the United States (Number of years)?

______________ Years

5. How long have you lived in Chicago (Number of years)?

______________ Years

6. Have you travelled internationally?

Yes No

If so, when and where and how long were you abroad?

7. To your knowledge, have you ever been treated with an antiparasitic medication?

Yes No

If so, when, and with what medication and when were you treated?

8. Which of the following categories best represents your household income:

____ < $20,000 per year
____ $20,000 - $40,000 per year
____ $40,000 - $60,000 per year
____ $60,000 - $ 100,000 per year
____ > $100,000 per year

9. What is the total number of years you have spent in school (example, if you completed high school the number would be 12, if you have a college degree the number would be 16)? _________

10. What medical problems have you been diagnosed with (examples, diabetes or hypertension)?

11. What medications do you take on a regular basis?

12. Do you have any medication allergies?

Yes No

If so, to what?

Do you have any seasonal allergies?

Yes No

13. Do you smoke?

Yes No

If yes, please list the number of cigarettes per day you smoke:

Please list how long you have smoked:

14. Do you drink alcohol on a regular basis?

Yes No

If yes, how many drinks do you consume per day?

Please list how many years you have been drinking alcohol regularly:

15. Are there any diseases that run in your family? If yes, describe.

Please check if you have experienced any of the following symptoms WITHIN THE PAST YEAR:

Symptoms related to your eyes*:*

____ Change in vision
____ Double vision
____ Loss of vision

Symptoms related to your Ears, Nose, Mouth, Throat*:*

____ Nosebleeds
____ Loss of hearing

Symptoms related to your skin:

____ Itching
____ Hives
____ Rash
____ A yellow color on your skin

Symptoms related to your stomach:

____ Heartburn
____ Difficulty swallowing
____ Pain when swallowing
____ Abdominal pain
____ Diarrhea
____ Swelling in your abdomen
____ Nausea/vomiting
____ Blood in your stool

Symptoms related to your lungs:
____ Wheezing
____ Shortness of Breath
____ Cough

Symptoms related to your heart:
____ Chest Pain or Pressure
____ Palpitations (pounding heart)
____ Irregular heart beat

Symptoms related to your muscles or joints*:*
____ Pain in your joints or muscles
____ Stiffness in your joints

Symptoms related to your nervous system:
____Denies dizziness
____ Numbness/tingling in your
hands/feet
____ Feelings of anxiety or depression

Other physical complaints:
____ Weight loss
____ Headache
____ Swelling in your legs
____ Blood in the urine
____ Pain when urinating
____ Increased urination
____ Fatigue
____ Fevers
____ Weakness
____ Difficulty concentrating
____ Yellow color in your eyes

If you experienced any of the above symptoms, please describe:

Please check if you are experiencing any of the following symptoms RIGHT NOW:
Symptoms related to your eyes*:*
____ Change in vision
____ Double vision
____ Loss of vision

Symptoms related to your Ears, Nose, Mouth, Throat*:*
____ Nosebleeds
____ Loss of hearing

Symptoms related to your skin:
_____ Itching
_____ Hives
_____ Rash
_____ A yellow color on your skin

Symptoms related to your stomach:
_____ Heartburn
_____ Difficulty swallowing
_____ Pain when swallowing
_____ Abdominal pain
_____ Diarrhea
_____ Swelling in your abdomen
_____ Nausea/vomiting
_____ Blood in your stool

Symptoms related to your lungs:
_____ Wheezing
_____ Shortness of Breath
_____ Cough

Symptoms related to your heart:
_____ Chest Pain or Pressure
_____ Palpitations (pounding heart)
_____ Irregular heart beat

Symptoms related to your muscles or joints*:*
_____ Pain in your joints or muscles
_____ Stiffness in your joints

Symptoms related to your nervous system:
_____Dizziness
_____ Numbness/tingling in your hands/feet
_____ Feelings of anxiety or depression

Other physical complaints:
_____ Weight loss
_____ Headache
_____ Swelling in your legs
_____ Blood in the urine
_____ Pain when urinating
_____ Increased urination
_____ Fatigue
_____ Fevers

____ Weakness
____ Difficulty concentrating
____ Yellow color in your eyes

If you experienced any of the above symptoms, please describe:

Please indicate if you have EVER experienced the following symptoms AT ANY TIME IN YOUR LIFE:

____ Seizure. If yes, please explain.

____ Loss of consciousness. If yes, please explain.

____ Worms in your stool. If yes, please explain.

Cuestionario de estudio.

Por favor conteste las siguientes preguntas.

1. ¿Cuál es tu edad? ___ años

2. ¿Cuál es tu género: ___ masculino ___ femenino

3. ¿Dónde naciste (ciudad y país)? País: ____________

Ciudad: ____________

Para las siguientes preguntas, por favor circule la respuesta correcta.

3.a. ¿Se crió en un sitio urbano o rural?	Urbano	Rural
1. ¿Recuerdas a beber agua de pozo-como un niño?	Sí	No
3.c. ¿Vivía en una casa con techo de paja?	Sí	No

3.d. ¿Pasaste una gran cantidad de tiempo alrededor de los animales como un niño?

Sí No

Si es así, que los animales (por favor lista):

3.e. ¿Te acuerdas de bañarse en arroyos o estanques como un niño?

Sí No

3.f. ¿Recuerda con frecuencia caminar descalzo como un niño?

Sí No

4. ¿Cuánto tiempo llevas en los Estados Unidos (varios años)?

___ Años

5. ¿Cuánto tiempo llevas en Chicago (varios años)?

___ Años

6. ¿Has viajado internacionalmente? Sí No

¿Si es así, cuándo y dónde y cuánto tiempo estuviste en el extranjero?

7. A su conocimiento, ¿has sido tratado con un medicamento antiparasitario?

Sí No

¿Si es así, cuándo y con qué medicamentos y cuando trataron?

8. ¿Cuál de las siguientes categorías mejores representa el ingreso de su hogar:

___ < $20.000 por año
___ $20.000 - $40.000 por año
___ $40.000 - $60.000 anuales
___ $60.000 - $ 100.000 por año
___ > $100.000 por año

9. ¿Cuál es el total número de años que ha pasado en la escuela (por ejemplo, si terminado la escuela secundaria sería el número 12, si tienes un título universitario el número sería 16)? _________

10. ¿Qué problemas médicos ha sido diagnosticados con (ejemplos, la diabetes o la hipertensión).

11. ¿Qué medicamentos toma sobre una base regular?

12. ¿Tiene alguna alergia medicamentos?

Sí No

Si es así, ¿a qué?

¿Tienes alergias estacionales?

Sí No

13. ¿Fuma usted?

Sí No

En caso afirmativo, por favor enumere el número de cigarrillos fumados por día que fumas:

Por favor indique cuánto ha fumado:

14. ¿Bebes alcohol con regularidad?

Sí No

En caso afirmativo, ¿Cuántas copas consume por día?

Por favor indique cuántos años usted ha estado bebiendo alcohol regularmente:

15. ¿hay alguna enfermedad que se ejecutan en tu familia? En caso afirmativo, describir.

Por favor, compruebe si ha experimentado cualquiera de los siguientes síntomas durante el año pasado:

Síntomas relacionados con tus ojos*:*
___ El cambio en la visión
___ Visión doble
___ Pérdida de la visión

Síntomas relacionados con tus oídos, nariz, boca, garganta*:*
___ Hemorragias nasales
___ Pérdida de la audición

Síntomas relacionados con la piel:
___ Picazón
___ Colmenas
___ Erupción
___ Un color amarillento en la piel

Síntomas relacionados con el estómago:
___ Ardor de estómago
___ Dificultad para deglutir
___ Dolor al tragar

___ Dolor abdominal
___ Diarrea
___ Inflamación en el abdomen
___ Náuseas/vómitos
___ Sangre en las heces

Síntomas relacionados con los pulmones:
___ Sibilancias
___ Dificultad respiratoria.
___ Tos

Síntomas relacionados con el corazón:
___ Presión o dolor en el pecho
___ Palpitaciones (corazón latiendo)
Vencer a ___ irregulares del corazón

Síntomas relacionados con sus músculos o articulaciones*:*
___ Dolor en las articulaciones o músculos
___ Rigidez en las articulaciones

Síntomas relacionados con el sistema nervioso:
____Niega mareos
___ Entumecimiento/hormigueo en su manos/pies
___ Sentimientos de ansiedad o depresión

Otras quejas físicas:
___ La pérdida de peso
___ Dolor de cabeza
___ Hinchazón en las piernas
___ Sangre en la orina
___ Dolor al orinar
___ Aumentada la micción
___ Fatiga
___ Fiebres
___ Debilidad
___ Dificultad para concentrarse
___ Amarillo color en tus ojos

Si has experimentado cualquiera de los síntomas anteriores, por favor describa:

Compruebe por favor si usted está experimentando cualquiera de los siguientes síntomas ahora:

Síntomas relacionados con tus ojos*:*
___ El cambio en la visión

___ Visión doble
___ Pérdida de la visión

Síntomas relacionados con tus oídos, nariz, boca, garganta*:*
___ Hemorragias nasales
___ Pérdida de la audición

Síntomas relacionados con la piel:
___ Picazón
___ Colmenas
___ Erupción
___ Un color amarillento en la piel

Síntomas relacionados con el estómago:
___ Ardor de estómago
___ Dificultad para deglutir
___ Dolor al tragar
___ Dolor abdominal
___ Diarrea
___ Inflamación en el abdomen
___ Náuseas/vómitos
___ Sangre en las heces

Síntomas relacionados con los pulmones:
___ Sibilancias
___ Dificultad respiratoria.
___ Tos

Síntomas relacionados con el corazón:
___ Presión o dolor en el pecho
___ Palpitaciones (corazón latiendo)
___ Pulso irregular

Síntomas relacionados con sus músculos o articulaciones*:*
___ Dolor en las articulaciones o músculos
___ Rigidez en las articulaciones

Síntomas relacionados con el sistema nervioso:
____Mareo
___ Entumecimiento/hormigueo en su manos/pies
___ Sentimientos de ansiedad o depresión

Otras quejas físicas:
___ La pérdida de peso
___ Dolor de cabeza
___ Hinchazón en las piernas

___ Sangre en la orina
___ Dolor al orinar
___ Aumentada la micción
___ Fatiga
___ Fiebres
___ Debilidad
___ Dificultad para concentrarse
___ Amarillo color en tus ojos

Si has experimentado cualquiera de los síntomas anteriores, por favor describa:

Por favor, indique si alguna vez ha experimentado los siguientes síntomas en cualquier momento en su vida:
___ Convulsión. En caso afirmativo, por favor explique.

___ La pérdida de la conciencia. En caso afirmativo, por favor explique.

___ Gusanos en las heces. En caso afirmativo, por favor explique.
