# Supplementary material for: Parasitic infections represent a significant health threat among recent immigrants in Chicago
Source: Parasitol Res. 2020 Feb 1;119(3):1139–48. doi: 10.1007/s00436-020-06608-4 (PMC7075846; doi:10.1007/s00436-020-06608-4)
Supplement: Supplementary file 3 — (PDF 19 kb) [file 436_2020_6608_MOESM3_ESM.pdf]

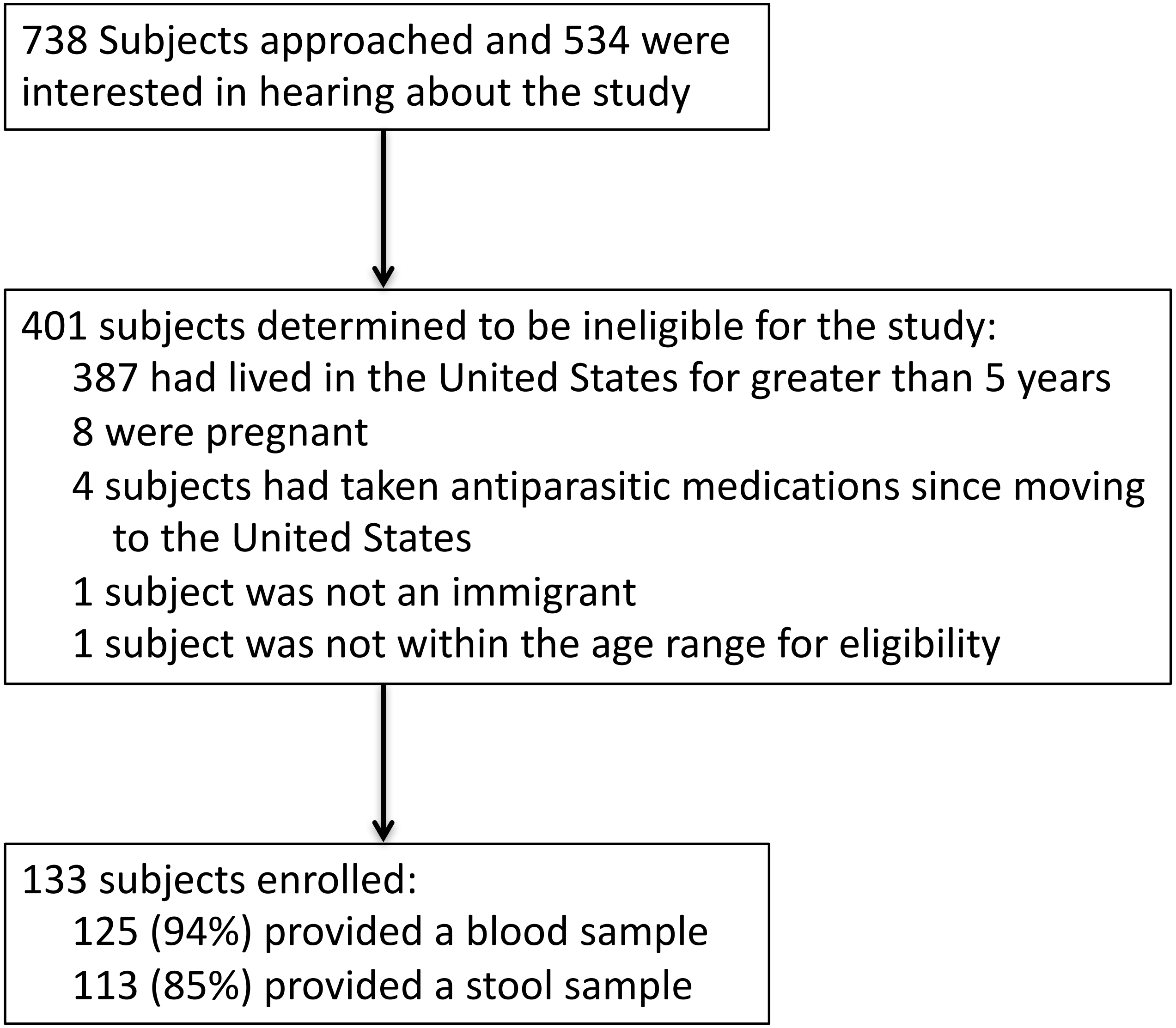
738 Subjects approached and 534 were interested in hearing about the study
401 subjects determined to be ineligible for the study:
387 had lived in the United States for greater than 5 years
8 were pregnant
4 subjects had taken antiparasitic medications since moving to the United States
1 subject was not an immigrant
1 subject was not within the age range for eligibility
133 subjects enrolled:
125 (94%) provided a blood sample
113 (85%) provided a stool sample
